# Performance of Ground Granulated Blast-Furnace Slag and Coal Fly Ash Ternary Portland Cements Exposed to Natural Carbonation

**DOI:** 10.3390/ma14123239

**Published:** 2021-06-11

**Authors:** Rosa Abnelia Rivera, Miguel Ángel Sanjuán, Domingo Alfonso Martín, Jorge Luis Costafreda

**Affiliations:** 1 Department of Cement Chemical Testing, LOEMCO, Universidad Politécnica de Madrid (UPM), 28906 Madrid, Spain; rosa.rcornelio@alumnos.upm.es; 2 Department of Science and Technology of Building Materials, Civil Engineering School, Technical University of Madrid, 28040 Madrid, Spain; 3 Mine and Energy Engineering School, Technical University of Madrid (UPM), 28003 Madrid, Spain; domingoalfonso.martin@upm.es (D.A.M.); jorgeluis.costafreda@upm.es (J.L.C.)

**Keywords:** cementitious materials, cement replacement materials, multicomponent binders, ternary cements, industrial by-products in cementitious mixes, coal fly ash, granulated blast-furnace slag, properties of cementitious materials, carbonation

## Abstract

Ternary Portland cements are new cementitious materials that contain different amounts of cement replacements. Ternary Portland cements composed of granulated blast-furnace slag (GBFS), coal fly ash (CFA), and clinker (K) can afford some environmental advantages by lowering the Portland cement clinker use. Accordingly, this is an opportunity to reduce carbon dioxide emissions and achieve net-zero carbon emissions by 2050. Furthermore, GBFS and CFA possess pozzolanic properties and enhance the mechanical strength and durability at later ages. Compressive strength and natural carbonation tests were performed in mortar and concrete. Cement-based materials made with GBFS and/or CFA presented a delay in the compressive strength development. In addition, they exhibited lower carbonation resistance than that of mortar and concrete made with plain Portland cements. Concrete reinforcement remains passive in common conditions; however, it could be corroded if the concrete pore solution pH drops due to the carbonation process. Service life estimation was performed for the ternary cements regarding the carbonation process. This information can be useful to material and civil engineers in designing concretes made with these ternary cements.

## 1. Introduction

Ternary cements are binders composed of Portland cement clinker and two other components, which are usually blended at the cement mill. Ground granulated blast-furnace slag and siliceous coal fly ash are two major constituents in Portland cements due to their good durable properties in aggressive environments. By contrast, these cements carbonate faster than plain Portland cement [1]. This constitutes a major drawback: it means that the expected service life of concretes made with cements containing ground granulated blast-furnace slag and/or siliceous coal fly ash would be lower. Accordingly, this aspect should be considered in the decision of using these new cements. Furthermore, they are sensitive to wet-curing time with regard to the natural carbonation resistance [1]. 

Some studies on ternary cements made with coal ash and rice husk ash [2] or made with limestone mixed with ground granulated blast-furnace slag [3], coal fly ash [4,5], or natural pozzolans [6] were recently reported in several papers. Compressive strength enhancements were found in ternary systems made with silica fume and coal fly ash [7] or ground granulated blast-furnace slag [8].

Similar studies were performed with ground granulated blast-furnace slag and coal fly ash [9,10]; when CFA and GBFS were finely ground (6000 cm^2^/g), the compressive strength gain for all ages was improved [11]. By contrast, Jeong et al. [12] did not find any improvement in the mechanical performance. The final characteristics of the ternary cement systems is achieved as result of the combination of the individual characteristics of all the cementitious constituents and their synergy.

The cement industry is a significant carbon dioxide emitter mainly due to the calcination process and combustion of fuels [13]. Therefore, the use of ternary cements is being promoted to achieve the target of reducing carbon dioxide emissions to 50–55% below 1990 levels by 2030 and net-zero carbon emissions by 2050 [14], according to most of the proposed cement industry roadmaps for moving to a low carbon economy in 2050. 

### 1.1. Steel Reinforced Concrete Corrosion by Carbonation

Steel reinforced concrete structures must be durable to ensure the intended service life is reached [15]. The steel reinforcement is passivated by the high alkalinity achieved by the Portland cement hydration products. However, the passive state of the steel reinforcement might be ended by the reduction in the pH of the concrete pore solution due to the carbonation and/or the chloride attack.

Accordingly, reinforcement corrosion induced by carbonation plays a key role in the reinforced concrete structure’s service life, primarily when it is coupled with chloride attack [16], which is a major concern due to the risk of reinforced concrete structural failure [17]. Carbonation is a process described below.

### 1.2. Carbonation Chemical Process

The physico-chemical process known as carbonation consists of the reaction of carbon dioxide (CO_2_) with calcium phases (Ca^2+^) and water (H_2_O) to form calcium carbonate in Portland cement paste. Furthermore, carbon dioxide (CO_2_) can produce other carbonates depending on the reacting species [18].

The early stage begins with the dissolution of the carbon dioxide (CO_2_) in water (H_2_O) in terms of the Equation (1) and the dissolution of Ca(OH)_2_ according to Equation (2). Afterwards, carbon dioxide reacts primarily with calcium hydroxide (Ca(OH)_2_) and other calcium phases [19]. Calcium hydroxide initially reacts with carbon dioxide faster than C–S–H gel, as given in Equations (3) and (4) [20]. Calcium ion removal from the C–S–H gel leads to the formation of calcite, vaterite, aragonite, and other calcium carbonate polymorphs [19], and silica gel [21]. Therefore, the Ca/Si ratio drops depending on the initial Ca/Si ratio in the C–S–H gel, i.e., the greater Ca/Si ratio, the minor C–S–H gel carbonation rate [21].

CO_2_ (g) + H_2_O (aq) → CO_2_ (aq) + H_2_O (aq) (1)

Ca(OH)_2_ (s) + H_2_O (aq) → Ca(OH)_2_ (aq) + H_2_O (aq) (2)

Ca(OH)_2_ (aq) + CO_2_ (aq) → CaCO_3_ (s) + H_2_O (aq) (3)

(CaO)_x_(SiO_2_)(H_2_O) (s) + xCO_2_ (aq) → xCaCO_3_ (s) + SiO_2_(H_2_O)_y_ (s) + (z − y) H_2_O (aq) (4)

Carbonation of cements made with coal fly ash or ground granulated blast-furnace slag has some peculiar characteristics. Due to the pozzolanic reaction, Ca(OH)_2_ is partially consumed, leading to a C–S–H gel formation [22] with a lower Ca/Si ratio. By contrast, a lower permeability was reported in blended pastes [23]. In general, these cements have a poor carbonation resistance, primarily in short curing periods [24]. Consequently, it is of great importance to define an adequate concrete mix design with ternary cements, made with coal fly ash and ground granulated blast-furnace slag, to be used in reinforced concrete structures with guarantees regarding the required service life.

Wrapping up, while carbonation is being developed, the microstructure becomes denser, and the pore solution pH lowers.

### 1.3. Carbonation Kinetic Mechanism

Different models were proposed to outline the cement paste carbonation process [16,20,25,26]. There are some descriptions of natural carbonation process that civil and material engineers use to make service life predictions for reinforced concrete structures. Some of them are based on the chemistry involved in cement paste carbonation [25], but more complicated models were also developed [26]. The drawback of these models is that they are too complex. A good model is both as simple and as accurate as possible, making it easy to understand.

This physico-chemical process primarily proceeds by diffusion and the carbonation depth is normally used to calculate the carbonation coefficient, B, as shown in Equation (5) [27], and the CO_2_ diffusion coefficient, D, given in Equation (6).

(5) $$x=B\sqrt{t}$$

where:

*B* = carbonation coefficient (mm/year^0.5^)
*x* = carbonation depth (mm)
*t* = natural carbonation exposure time (year)

(6) CX−C1C2−CX=Πxt2Dexpx2t4Derfxt2D≈C1CX

where:

*C_x_* = CO_2_ concentration at discontinuity (kmol/m^3^)
*C*_1_ = CO_2_ concentration in surroundings (kmol/m^3^)
*C*_2_ = CO_2_ concentration in the cement paste (kmol/m^3^)
*D* = Diffusion coefficient of the CO_2_ (m^2^/s)

Both parameters, B and D, are assumed to be constant (Table 1), but they are dependent on binder composition, environmental relative humidity, hydration degree, CO_2_ concentration, and pore size distribution (PSD), among other factors.

The European standards series known as Eurocodes specifies how structural design in buildings and civil engineering works should be conducted in Europe (EU). In Eurocode 2: Design of concrete structures (EN 1992), Part 1-1: General rules, and rules for buildings (EN 1992-1-1) [28], describes the requirements for serviceability, safety, and durability of reinforced concrete structures, including the limit state concept [28].

This standard considers some basic requirements regarding CO_2_ diffusion and concrete cover. Four carbonation exposure classes (XC) and a design service life of 100 years are defined (as illustrated in Table 2).

Concrete cover protects the reinforcement from carbonation induced corrosion initiation. Then, the minimum concrete cover depth required to be provided for corrosion against carbonation according to Eurocode 2 is given in Table 2.

The aim of this work was to assess the performance of ternary Portland cements composed of granulated blast-furnace slag (GBFS), coal fly ash (CFA), and clinker (K) with regard to their carbonation resistance and mechanical strength development. Natural carbonation testing on ternary cement concretes will be used to assess the potential for improvement of this material. Accordingly, the minimum concrete cover required to prevent corrosion against carbonation given by the Eurocode 2 will be taken as reference value. These results will be useful to material and civil engineers in designing reinforced concrete made with ternary cements.

## 2. Materials and Methods

### 2.1. Materials

The raw materials used to prepare the ternary cements were a Portland cement CEM I 42.5 R—EN 197-1 [29], ground granulated blast-furnace slag and coal siliceous fly ash, which chemical compositions are shown in Table 3. Chemical determination of SiO_2_, Al_2_O_3_, Fe_2_O_3_, CaO, MgO, SO_3_, Na_2_O, K_2_O, loss on ignition (LOI), insoluble residue (IR), and Cl^−^ was conducted following the methodology of the European standard for chemical analysis of cement, i.e., EN 196-2 [30].

Free lime content in the Portland cement and coal fly ash were 1.31% and 0.5%, respectively. Reactive calcium oxide and reactive silica amount in the ground granulated blast-furnace slag were 3.84% and 44.78%, respectively. 

Portland cement (CEM I 42.5 R) and coal fly ash (VA) fineness measured according to EN 196-6 [31] were 3246 cm^2^/g and 3772 cm^2^/g, respectively, whereas their densities were 3.12 g/cm^3^ and 2.4 g/cm^3^, respectively. The granulated blast-furnace slag was ground in a ball mill to reach two fineness values of 3489 cm^2^/g (SA) and 4630 cm^2^/g (SB). 

In conclusion, coal fly ash and ground granulated blast-furnace slag fulfill the general requirements set out by the European standard EN 197-1 regarding the characteristics of the cement constituents.

### 2.2. Ternary Cements Composition and Mix Design

Ternary cements prepared for this study were manufactured by blending of coal fly ash (V), ground granulated blast-furnace slag (S), and Portland cement (CEM I 42.5 R) in the percentages presented in Table 4 to form eight different ternary cements. The Portland cement was utilized as reference (100%).

Both additions were provided by Cementos Tudela Veguín, S. A. (Aboño, Spain), and the Portland cement by LafargeHolcim (Villaluenga de la Sagra, Spain).

A high range water reducing (superplasticizing) admixture for concrete was provided by Sika España, Madrid, Spain (Sika ViscoCrete-20 HE).

#### 2.2.1. Mortar Mix Design

Mortar mixes were elaborated by blending blast-furnace slag and coal siliceous fly ash contents of 25% or 40%. Accordingly, CEM I 42.5 R percentages were 20%, 35%, or 50% depending on each individual case (as illustrated in Table 4). 

Prismatic mortar specimens (40 × 40 × 160 mm) were produced with CEN sand and distilled water following the methodology defined by the European standard EN 196-1 [32]. Therefore, the water/cement ratio was 0.50 and the cement/sand ratio was 1/3. Mortar specimens were demolded 24 ± 1 h after casting. Later, they were stored under lime saturated water for 2, 7, or 28-days.

#### 2.2.2. Concrete Mix Design

Two concrete mixes (as illustrated in Table 5) were chosen to assess the mechanical and carbonation performance of the ternary cements, as shown in Table 6.

### 2.3. Methods

#### 2.3.1. Compressive Strength

Concrete cylindrical specimens (Ø15 × 30 cm) were made and cured according to the European standard EN 12390-2:2019 [33] and tested for compressive strength at 28 days and 90 days according to EN 12390-3:2019 [34]. The average value calculated from two specimens was taken.

#### 2.3.2. Carbonation

The mortar and concrete specimens were tested for natural carbonation following the technical specification CEN/TS 12390-10 [35]. Accordingly, mortars and concretes were exposed to the natural outdoor environment under shelter from rain conditions (relative humidity of 60 ± 5%). 

Carbonation depth measurements were taken in the prism mortar specimens at 20 months of natural exposure, and at 12 months in the concrete Ø75 × 100 mm cylinders.

At the beginning of testing, samples were sawn up into 25 mm slices. It was assumed that the sawed cut is adequate for carbonation depth testing. Therefore, depth of carbonation was assessed on the freshly sawed area, which was cleaned of loose particles. The phenolphthalein indicator coloration was assessed visually in the laboratory. The cleaned surface was sprayed with a phenolphthalein indicator solution. In certain circumstances, when coloration was absent or very weak on the sprayed area, the surface was sprayed again after 20 min.

Carbonation depth readings were collected in four points of each mortar or concrete specimen. Later, carbonation coefficient, B, and CO_2_ diffusion coefficient, D, were calculated according to Equations (5) and (6), respectively.

Finally, concrete carbonation coefficients were used to estimate the design service life regarding reinforcing steel corrosion.

## 3. Results and Discussion

### 3.1. Compressive Strength

**Figure 1:**
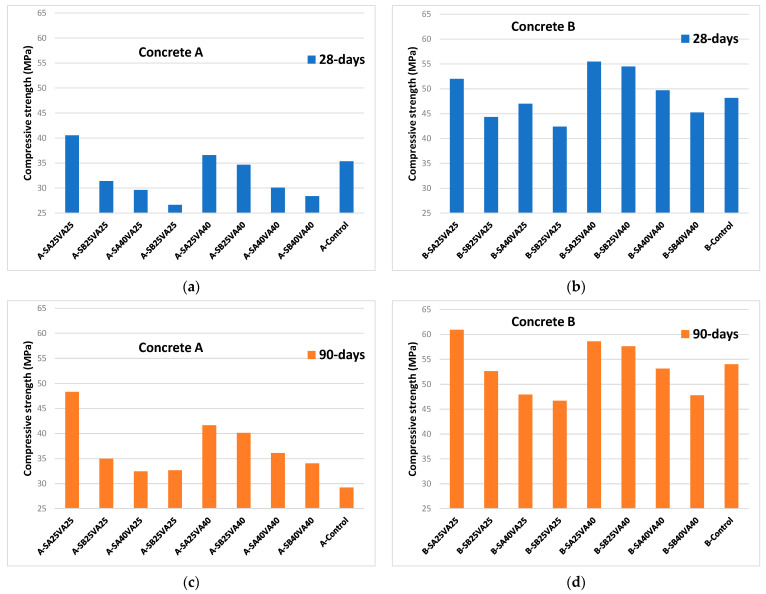
Compressive strength at 28- and 90-days (MPa): (**a**) concrete A at 28-days; (**b**) concrete B at 28-days; (**c**) concrete A series at 90-days, and (**d**) concrete B series at 90-days.

Compressive strength tests were performed in concretes A and B. The compressive strength of the concrete cylinder test can provide a general idea about all the characteristics of a concrete. By this easy test one judge that whether concrete mix design and execution was done properly or not. Figure 1 collects all the compressive strength at 28- and 90-days results obtained for concretes A and B.

Concrete mix A—control is a concrete grade C30/37 according to the European standard EN 206 [36]; Concrete mix B—control was a concrete grade C45/55. The minimum characteristic cylindrical strength at 28-days (f_ck_) for C30/37 concrete grade is 30 MPa, while for C45/55, concrete grade is 45 MPa.

The effect of the ground granulated blast-furnace slag fineness at 28-days is more evident in concrete mixes B than in concrete mixes A. The finer the GGBFS is, the higher the concrete compressive strength is (SA: 3489 cm^2^/g, SB: 4630 cm^2^/g). Furthermore, mixes B-SA25VA40, B-SB25VA40, and B-SA40VA40 presented higher 28-days concrete compressive strength results than that of the control concrete B. In addition, B-SA25VA25 mixture also had higher 28 days compressive strength than that of the control one. Concrete with a lower concrete grade does not exhibit similar improvements, i.e., only concrete mixes B-SA25VA25 and A-SA25VA40 had higher 28-days compressive strength.

Compressive strength data higher than the designed value could not be beneficial from a structural viewpoint because undue stiffness might cause an inadequate redistribution of internal forces and exhibit greater than projected stress and deflection [37].

90-days compressive strength results for concrete B series followed the same trend as for 28-days. Nevertheless, concrete A with additions developed a greater strength gain than that of the control concrete A without additions. In this case, mixes A-SA25VA40, A-SB25VA40, A-SA40VA40, and A-SA25VA25 exceeded the A-control level. Consequently, the positive effect of the additions in the lower strength class A concretes becomes apparent when the compressive strength is tested at 90-days.

Over longer periods (90-days) SA provides similar compressive strengths than SB (as illustrated in Figure 1). However, at an earlier period (28-days), this similarity was not found by the faster pozzolanic reaction development in the finer binder.

### 3.2. Carbonation

#### 3.2.1. Mortar Carbonation

**Figure 2:**
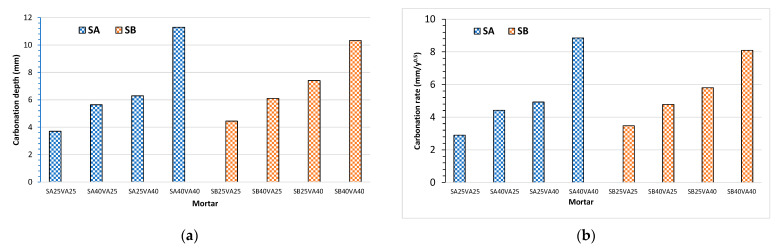
Carbonation resistance of mortar specimens made with ternary cements made with coal fly ash (V) and two ground granulated blast-furnace slags (SA: coarse and SB: fine) at 20 months of natural exposure: (**a**) carbonation depth measurements; (**b**) carbonation coefficients.

Mortar’s carbonation depth readings by the phenolphthalein method were collected at 20 months, and the average results were calculated with the readings of three mortar samples (as illustrated in Figure 2a) to obtain the carbonation coefficients given by Equation (5) (as illustrated in Figure 2b). As expected, the higher the addition content in the ternary cement, the deeper the carbonation depth [1,22,24]. The increase in carbonation depth may be due to the lower clinker content and, therefore, the lower alkaline reserve of the pore solution. Furthermore, the nature of the porosity and the alkaline reserve promoted by the cement hydration products are the major factors associated with the cement-based materials that affect carbonation.

The effect of the ground granulated blast-furnace slag (GGBFS) fineness on the carbonation depth is negligible at 25% GGBFS levels. However, with 40% of GGBFS, the finer ground granulated blast-furnace slag (GGBFS) presents a better carbonation resistance. This fact could be attributed to the lower porosity in this last case [22].

The effect of the partial replacement of coal fly ash in ternary cements decreasing the alkaline reserve and increasing the carbonation rate is lower than in the case of ground granulated blast-furnace slag (GGBFS) [38].

#### 3.2.2. Concrete Carbonation

**Figure 3:**
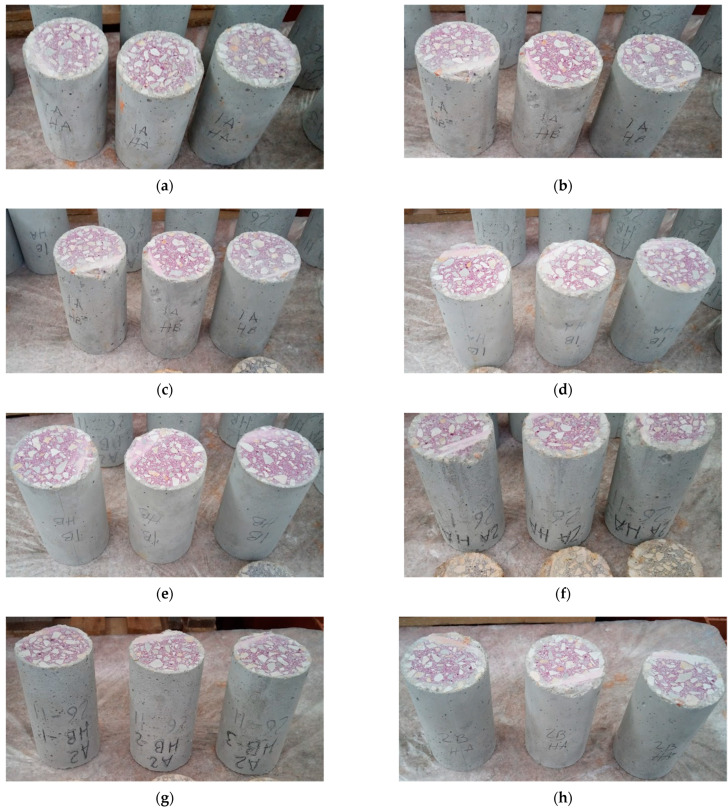

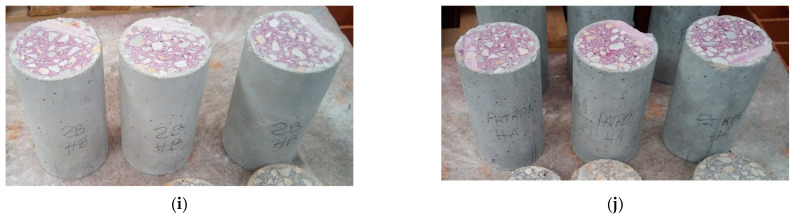
Carbonation depth results obtained for concretes A and B measured in three concrete samples: (**a**) A-SA25VA25; (**b**) B-SA25VA25; (**c**) A-SB25VA25; (**d**) B-SB25VA25; (**e**) A-SA40VA25; (**f**) B-SA40VA25; (**g**) B-SB40VA25; (**h**) B-SB40VA25; (**i**) reference concrete A; (**j**) reference concrete B.

**Figure 4:**
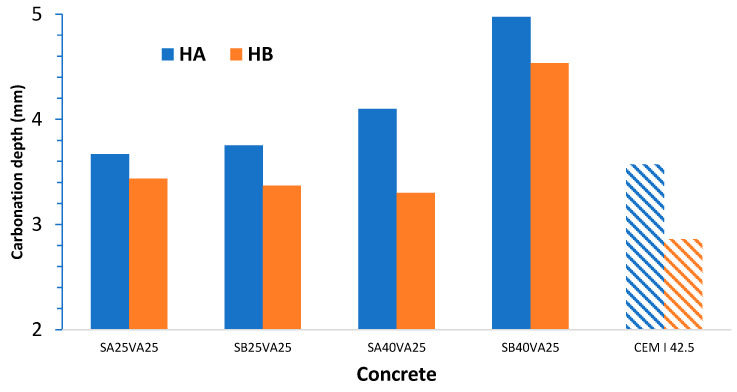
Carbonation depth of concrete samples at 12 months.

Carbonation depth results obtained for concretes A and B were calculated with the results of three concrete samples (as illustrated in Figure 3). The average values are shown in Figure 4.

The carbonation depth in concretes is smaller than that of mortars, but it is still significant. The carbonation fronts of the coal fly ash and GGBFS containing mortars were deeper (until 4–11 mm) than that of concretes (until 3–5 mm).

Concrete having the least amount of clinker (HA) presents the higher carbonation depth. In line with this result, concrete made without additions offers the best carbonation resistance.

In this case, the same as in the mortar’s case, the effect of the ground granulated blast-furnace slag (GGBFS) fineness on the carbonation depth is negligible at 25% GGBFS levels. By contrast, with 40% of GGBFS, the finer GGBFS presents a worse carbonation resistance. The fineness has an important bearing on the hydration rate, and hence, on the microstructural development and the strength gain. Finer GGBFS offers a greater surface area for reaction and, therefore, accelerates the microstructural development and its effect on the mechanical properties. Furthermore, the pozzolanic reaction of the GGBFS cement paste between reactive silica or alumina in the GGBFS particles and calcium hydroxide (Ca(OH)_2_) formed from cement hydration in the presence of water also accelerates lowering the alkaline reserve [38]. In addition, increasing the fineness of a cement also increases the amount of mixing water required to achieve a given consistency and could increase the porosity of the concrete.

**Figure 5:**
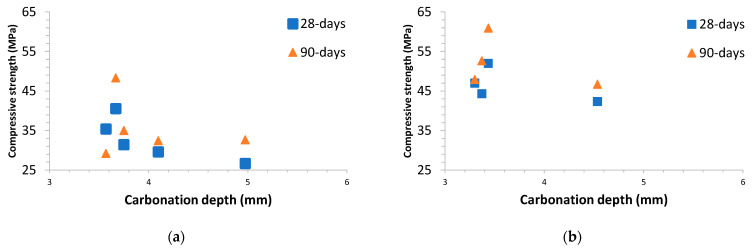
Carbonation depth at 12 months of natural exposure versus compressive strength at 28-days and 90-days: (**a**) concrete A; (**b**) concrete B.

Figure 5 shows the relationship between the carbonation depth at 12 months of natural exposure versus compressive strength at 28-days and 90-days for the concretes A and B. As expected, the higher the compressive strength is, the lower carbonation depth was obtained.

For concretes with a 28-days compressive strength lower than 45 MPa (concrete A), a clearer trend between the carbonation depth and the compressive strength at 28-days and 90-days was found. By contrast, mentioned correlation was not found in more dense and compact concretes, i.e., concretes with compressive strength at 28-days over 45 MPa (concrete B). In this last case, it would probably be necessary to have more testing time to achieve any correlation between these two parameters. In general, similar trends were reported in the literature [27]. Summing up, the high scatter found in the results prevents us from deducing causal relationships based on mechanical strength versus carbonation test measurements. Accordingly, compressive strength versus carbonation relationships can only be achieved through concretes made with the same constituents in different proportions.

### 3.3. Reinforced Concrete Structures Service Life Assessment

Concrete carbonation-induced steel corrosion is one of the major issues of the durability and service life for reinforced concrete (RC) structures in atmospheric environment. Since the rate of carbonation depends on the concrete quality, assessment of the carbonation resistance and the service life estimation of the new ternary cements are necessary.

**Figure 6:**
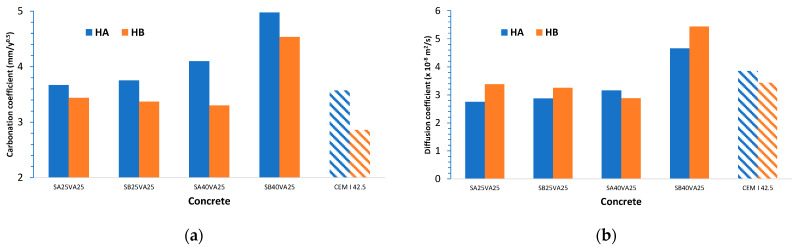
Input data for carbonation service life estimation for reinforced concrete structures exposed to atmospheric environment: (**a**) carbonation coefficient (mm/year^0.5^); (**b**) carbonation diffusion coefficient (×10^−8^ m^2^/s).

**Figure 7:**
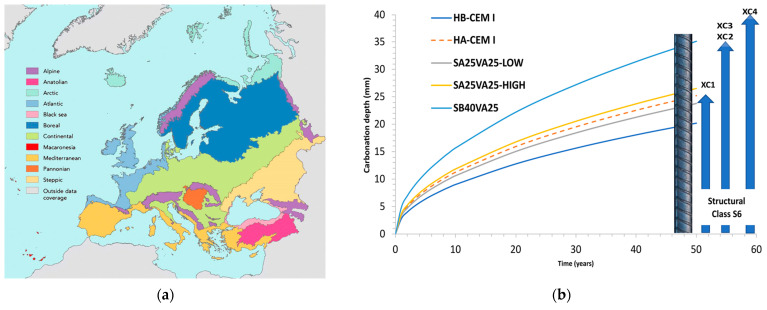
Carbonation-induced reinforcement corrosion estimation: (**a**) biogeographical regions in Europe to consider exposure class (XC1—XC4); (**b**) carbonation depths for ternary cements.

The carbonation rate is inversely proportional to the square root of the age of the reinforced concrete structure, as shown in Equation (5) [27]. Accordingly, the carbonation coefficient, B, can be calculated from the carbonation depth readings (as illustrated in Figure 6a). Afterwards, this value can be used to estimate the reinforced concrete structures’ service life (as illustrated in Figure 7). In addition, the carbonation diffusion coefficient, D, was calculated following Equation (6). This second approach yielded similar results, but it was more time consuming (as illustrated in Figure 6b).

Concrete with a larger quantity of GGBFS has a higher carbonation coefficient, particularly in the case of the finer GGBFS. Accordingly, a shorter service life period for reinforced concrete structures exposed to atmospheric environment can be expected.

Carbonation diffusion coefficients were quite similar for concretes A and B made with the ternary cements SA25VA25, SB25VA25, and SA40VA25 (between 2.75 × 10^−8^ and 3.38 × 10^−8^), which are slightly lower than those of the reference concretes A and B made with CEM I 42.5.

The choice of adequately durable concrete for carbonation-induced reinforcement corrosion protection requires consideration of the composition of concrete. Accordingly, this may result in a higher concrete compressive strength than is required for structural design. The relationship between concrete strength classes and corrosion induced by carbonation exposure class is described by three indicative strength classes in the European standard EN 206 [36], i.e., C20/25 for XC1, C25/30 for XC2, and C30/37 for XC3 and XC4. According to Figure 1, all the concretes are above 25 MPa, so they are suitable for use in XC1 exposure class. Furthermore, concrete B manufactured with ternary cements is adequate for XC2—XC4 exposure classes (as illustrated in Table 2).

Natural carbonation estimation is necessary to evaluate the ternary cement compositions and the effect of the blast-furnace slag fineness to perform an accurate service life design estimation. Carbonation-induced reinforcement corrosion process may be divided into two steps called initiation and propagation periods [39]. The initiation period is the time until the steel reinforcement becomes depassivated when the concrete cover is carbonated. This first period is modelled by Equation (5) [27], and the predicted carbonation depth at the end of the required service life should be lower than the concrete cover. This information will help civil engineers to estimate service life in case of carbonation-induced corrosion.

Carbonation depths at 50 years were calculated from the carbonation coefficients derived after one year of exposure for two qualities of concrete containing different types of ternary cements and exposed to a natural atmosphere containing 0.03% CO_2_ (as illustrated in Table 7). To assess whether concrete carbonation-induced steel corrosion is a risk for the reinforced concrete structure, these results are compared to that of the minimum cover thickness required by the Eurocode 2 [28] for XC environments (as illustrated in Table 2). The minimum concrete cover varies from 10 to 40 mm for the 6 Structural Classes.

Because natural carbonation testing was performed at 60% RH, sheltered from rain, XC3 corrosion induced by carbonation exposure class is considered in the present analysis (Prescriptive or Deemed to Satisfy rules). Accordingly, an S4 structural class requires a minimum cover thickness of 25 mm (as illustrated in Table 2). This concrete cover is enough for concrete B and cements SA25VA25, SB25VA25, SA40VA25, and CEM I 42.5. By contrast, all the concretes type A presented carbonation depths above 25 mm after 50 years. In this case, the same types of ternary cements comply with the requirements set out in the specification for S5 structural class. However, SB40VA25 ternary cement can be used for concrete B and S6 structural class.

Furthermore, reinforced concrete service life could be increased by using some finishing materials such as paints [40] and surface protective materials modified with nano-SiO_2_ [41].

Normally, the durability design of reinforced concrete structures uses a concept in which the performance is assessed with deemed-to-satisfy rules (concrete mix design and concrete cover) based on experience. This concept works well for traditional materials for which long experience is available; nevertheless, new additions need the assessment based on performance testing.

Currently, exposure resistance classes (ERC) are proposed to classify reinforced concrete with respect to resistance against corrosion induced by carbonation (XRC class) following a performance-based concrete approach. The selection of concrete to resist deterioration and protect against corrosion for this exposure class is based on the exposure resistance classes given in EN 206 [28,36]. Table 8 shows the minimum cover specified for each exposure resistance class (ERC) within the XC3 exposure class. All the concretes type B can be used for XRC 4–XRC 7, except concrete made with the SB40VA25 ternary cement. Conversely, type A concretes can be used only for XRC 6 and XRC 7 (as illustrated in Figure 7). In addition, a maximum mean value of the carbonation coefficient for mentioned exposure resistance classes (ERC) is shown in Table 8.

The carbonation coefficient is above 2.7 mm/y^0.5^ for all the concretes. Therefore, they can only be used in XRC 4–XRC 7 exposure resistance classes (ERC). This result is in line with the previous analysis.

These concretes may be applied in wide range of applications, such as reinforced concrete exposed to marine environment in harbours, building foundations, concrete pipes, self-compacting concrete, and so on. By contrast, it is not recommended for prestressed concrete.

Furthermore, carbonation is considered by the cement sector as a way to achieve carbon neutrality by 2050 [3]. Therefore, carbon dioxide uptake by mortars and concretes should be considered in the climatic models included in the IPCC’s Assessment Reports. Appropriate technical measures for future concrete mix design should not only consider durable and sustainable aspects, i.e., to ensure the reinforced concrete service life, but also carbon dioxide uptake.

## 4. Conclusions

Concrete carbonation is a physico-chemical process influenced by the cement type. Data presented in this paper allow the drawing of meaningful and practical technical conclusions regarding the ternary cements used. However, such conclusions, as with any generalization, should be read with caution since results may change with variations in concrete mix design. Conclusions are listed next.

- The use of GGBFS with coal fly ash, both as additions or as a partial replacement of Portland cement, results in the depletion of alkaline supply and, therefore, an increase in the carbonation rate.
- Ternary cements reduce compressive strength at 28-days of lower strength class concretes (C30). On the other hand, it sharply increases at 90 days. By contrast, concrete type B with a higher compressive strength exhibited a lower strength gain at 90-days. However, the effect of the additional content on the reduction of the compressive strength is more evident.
- There is an inverse relationship between the clinker content and concrete carbonation rate. The effect of the GGBFS fineness on the carbonation depth is negligible at 25% levels. Nevertheless, at 40% levels, the finer GGBFS showed better carbonation resistance due to a denser microstructure in this last case.
- Based on the carbonation and diffusion coefficients, the carbonation process is apparently better related with the alkaline reserve calculated by using the carbonation diffusion coefficient than the carbonation coefficient since it considers the CaO content in the calculations.
- Service life estimation related to carbonation was performed depending on the ternary cement used in the concrete mix. The ternary cement with the highest amount of blast-furnace slag yielded the lowest reinforced concrete service life.

All the above results should be considered by the civil engineers to design civil and building structures made of concrete with GGBFS and coal fly ash ternary cements.

## Figures and Tables

**Table 1:** Usual values of B and D [27].

	Low Quality	Average Quality	Good Quality
**B (mm/y^0.5^)**	>9	5–9	<5
**D (× 10^−7^ m^2^/s)**	>4	0.5–4	0.5

**Table 2:** Minimum concrete cover depth (mm) for corrosion induced by carbonation.

Environmental Requirement for Minimum Concrete Cover Depth (mm)	Structural Class					
Exposure Class: Corrosion Induced by Carbonation	S1	S2	S3	S4	S5	S6
XC1—Dry or permanently wet	10	10	10	15	20	25
XC2—Wet, rarely dry	10	15	20	25	30	35
XC3—Moderate humidity	10	15	20	25	30	35
XC4—Cyclic wet and dry	15	20	25	30	35	40

**Table 3:** Chemical compositions of ground granulated blast-furnace slag, GGBFS, coal siliceous fly ash, CFA, and Portland cement, CEM I 42.5 R, determined according to EN 196-2 (%).

Constituent	SiO_2_	Al_2_O_3_	Fe_2_O_3_	CaO	MgO	SO_3_	Na_2_O	K_2_O	LOI	IR ^1^	Cl^−^
CEM I 42.5 R	20.51	4.30	3.01	60.38	3.61	3.14	0.16	0.81	2.78	1.44	0.05
GGBFS	35.96	10.61	0.40	42.89	7.10	2.02	0.30	0.46	0.00	–	–
CFA	53.79	19.54	10.20	4.44	1.83	0.84	2.03	1.83	1.73	17.41	–

^1^ Insoluble residue measured by Na_2_CO_3_ method (EN 196-2) [30].

**Table 4:** Codes and proportions of ternary cements.

Code	Cement (%)	S (%)	V (%)	S—Fineness (cm^2^/g)
SA0VA0	100	0	0	–
SA25VA25	50	25	25	3489
SA40VA25	35	40	25	3489
SA25VA40	35	25	40	3489
SA40VA40	20	40	40	3489
SB25VA25	50	25	25	4630
SB40VA25	35	40	25	4630
SB25VA40	35	25	40	4630
SB40VA40	20	40	40	4630

**Table 5:** Concrete mix design for each concrete, A & B (kg/m^3^).

Component	Ternary Cement	Sand	Gravel	Water	Additive ^1^
Concrete A (kg/m^3^)	250	880	1100	172	5.0
Concrete B (kg/m^3^)	350	840	1100	172	5.0

^1^ Sika ViscoCrete-20 HE.

**Table 6:** Ternary cements composition for each concrete, A & B (kg/m^3^).

Codification	Concrete A				Concrete B			
	CEM I	SA	SB	VA	CEM I	SA	SB	VA
Control	250				350			
SA25VA25	125	62.5		62.5	175	87.5		87.5
SB25VA25	125		62.5	62.5	175		87.5	87.5
SA40VA25	87.5	100		62.5	122.5	140		87.5
SB40VA25	87.5		100	62.5	122.5		140	87.5
SA25VA40	87.5	62.5		100	122.5	87.5		140
SB25VA40	87.5		62.5	100	122.5		87.5	140
SA40VA40	50	100		100				
SB40VA40	50		100	100				

**Table 7:** Carbonation depth (mm) estimated for 50 years (XC3 environment defined in Table 2).

Concrete Type	SA25VA25	SB25VA25	SA40VA25	SB40VA25	CEM I 42.5
**HA**	25.9	26.5	29.0	35.2	25.2
**HB**	24.3	23.8	23.3	32.1	20.2

**Table 8:** Concrete cover (mm) and maximum mean value of carbonation coefficient (mm/years^0.5^) required for exposure resistance classes (ERC) given for XC3 exposure class.

Exposure Resistance Classes (ERC)	XRC 0.5	XRC 1	XRC 2	XRC 3	XRC 4	XRC 5	XRC 6	XRC 7
**Cover (mm)**	10	10	15	20	25	25	35	40
**Maximum carbonation coefficient (mm/years^0.5^)**	0.6	1.2	2.4	2.7	3.6	4.5	5.4	6.4

## Data Availability

Data is contained within the article.
